# The GUARDIAN system-a GNSS upper atmospheric real-time disaster information and alert network

**DOI:** 10.1007/s10291-022-01365-6

**Published:** 2022-12-03

**Authors:** Léo Martire, Siddharth Krishnamoorthy, Panagiotis Vergados, Larry J. Romans, Béla Szilágyi, Xing Meng, Jeffrey L. Anderson, Attila Komjáthy, Yoaz E. Bar-Sever

**Affiliations:** 1 Jet Propulsion Laboratory, California Institute of Technology, 4800 Oak Grove Drive, Pasadena, CA 91109 USA; 2 National Center for Atmospheric Research, 1850 Table Mesa Drive, Boulder, CO 80305 USA

**Keywords:** GNSS data, Geophysical data, Oceans and water, Atmosphere

## Abstract

We introduce GUARDIAN, a near-real-time (NRT) ionospheric monitoring software for natural hazards warning. GUARDIAN’s ultimate goal is to use NRT total electronic content (TEC) time series to (1) allow users to explore ionospheric TEC perturbations due to natural and anthropogenic events on earth, (2) automatically detect those perturbations, and (3) characterize potential natural hazards. The main goal of GUARDIAN is to provide an augmentation to existing natural hazards early warning systems (EWS). This contribution focuses mainly on objective (1): collecting GNSS measurements in NRT, computing TEC time series, and displaying them on a public website (https://guardian.jpl.nasa.gov). We validate the time series obtained in NRT using well-established post-processing methods. Furthermore, we present an inverse modeling proof of concept to obtain tsunami wave parameters from TEC time series, contributing significantly to objective (3). Note that objectives (2) and (3) are only introduced here as parts of the general architecture, and are not currently operational. In its current implementation, the GUARDIAN system uses more than 70 GNSS ground stations distributed around the Pacific Ring of Fire, and monitoring four GNSS constellations (GPS, Galileo, BDS, and GLONASS). As of today, and to the best of our knowledge, GUARDIAN is the only software available and capable of providing multi-GNSS NRT TEC time series over the Pacific region to the general public and scientific community.

**Supplementary Information:**

The online version contains supplementary material available at 10.1007/s10291-022-01365-6.

## Introduction

Tsunamis are large oceanic surface waves, triggered by submarine events (such as earthquakes, landslides, and volcanic eruptions), and able to propagate thousands of kilometers with relatively little attenuation. Due to conservation of energy in the shallow depths, the height of tsunami waves is amplified, leading them to become catastrophic near coastlines. As shown by many tragic events, tsunamis can impose an immense human and economic cost (National Geophysical Data Center 2022), making a case for tsunami early warning systems to be the center of our research focus.

These efforts fall within broad international frameworks targeting disaster risk reduction. The International Union of Geodesy and Geophysics (IUGG) established a special commission on Geophysical Risk and Sustainability (IUGG GeoRisk), with the goal of fostering research on geophysical hazards and their mitigation measures. In particular, the IUGG’s Resolution IV following its 2015 General Assembly (https://www.iag-aig.org/doc/5d10c798a8a37.pdf) urges to use GNSS as enhancing method to tsunami early warning systems (EWS). Under the IUGG, the International Association of Geodesy (IAG) includes the Global Geodetic Observing System (GGOS), within which the Geohazards Focus Group aims to enhance GNSS-based Tsunami Early Warning Systems (GTEWS) in particular. Under the aegis of the United Nations, member countries also collaborate on the Sendai Framework for Disaster Risk Reduction, an agreement whose goal is to better understand disaster risks and to enhance disaster preparedness. Our work is a tightly linked to these initiatives, and we maintain close collaborations with them.

Global Navigation Satellite Systems (GNSS) use radio waves for positioning applications (e.g., precise point positioning). Due to the dispersive nature of the earth’s ionosphere, GNSS signals transmitted over two different carrier frequencies will exhibit phase and pseudorange differences directly proportional to the ionospheric Total Electron Content (TEC) along the signal path (see, e.g., Teunissen and Montenbruck (2017)). While being a complication for positioning, this difference provides a sensitive probe of the TEC along the GNSS signal path.

Significant displacements of air at the earth’s surface may produce vertically propagating atmospheric acoustic and gravity waves, which typically reach the ionosphere with an 8–40-min delay (Astafyeva 2019; Astafyeva et al. 2011; Thomas et al. 2018; Vergados et al. 2020). Such perturbations may be caused by a broad spectrum of sources: earthquakes, tsunamis, volcanic eruptions, thunderstorms, meteoroids, deep convection events, and a variety of anthropogenic events (explosions, rocket launches, etc.).

The study of tsunami-driven ionospheric perturbations predates the GNSS era (Najita et al. 1974), but the current dense global satellite coverage allows for more straightforward and systematic approaches to study the ionospheric impacts of natural hazards (Artru et al. 2005; Liu et al. 2019; Manta et al. 2020; Occhipinti 2015; Occhipinti et al. 2013). Other types of potentially destructive events may also perturb the ionosphere: earthquakes (Astafyeva et al. 2014; Heki 2021; Maletckii and Astafyeva 2021; Sanchez et al. 2022), volcanic eruptions (Astafyeva 2019; Astafyeva et al. 2022; Heki 2022; Manta et al. 2021; Matoza et al. 2022; Themens et al. 2022), deep convective events (Lay et al. 2015), or space weather effects (Afraimovich et al. 2001). Finally, it is to be noted that ionospheric monitoring is particularly valuable for regions with sparse ground coverage such as remote volcanic islands and polar regions.

**Fig. 1:**
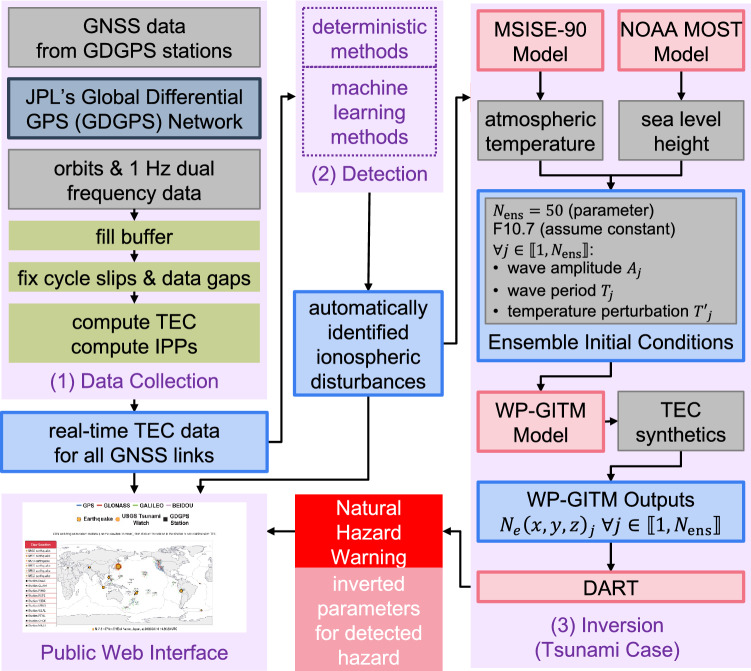
The GUARDIAN system architecture is composed of four main blocks, two of which are operational as of today and a third one of which has been validated as a standalone feature. We describe mainly block (1) and the Public Web Interface, which are the parts that are currently completely implemented and functional. Block (2) is not currently implemented in GUARDIAN; it will consist of an automated detection scheme (see the “Automated Detection” section). Block (3) is not currently implemented in GUARDIAN; it is an example inverse model for the retrieval of hazard parameters, in this case for tsunami waves, which has been validated as a standalone feature (see the “Tsunami Parameters Inversion” section). In block (3), the electron density ${N}_{e}$ is the direct product of the WP-GITM simulation (see the “Tsunami Parameters Inversion” section), and DART is the Data Assimilation Research Testbed (Anderson et al. 2009). See main text for detailed descriptions of each block. GNSS are Global Navigation Satellite Systems. JPL is NASA’s Jet Propulsion Laboratory. GDGPS is JPL’s Global Differential GPS network. IPPs are Ionospheric Pierce Points. TEC is the Total Electronic Content. For the public web interface, see https://guardian.jpl.nasa.gov

There exist several different EWS for tsunamis, all of which rely on ground-based or oceanic instruments and are subject to technical limitations. The Deep-ocean Assessment and Reporting of Tsunamis (DART–n.b., not to be confused with the DART in Fig. 1) buoys are deployed about 300 km away from coasts to measure changes in the water column height (Bernard and Meinig 2011; Meinig et al. 2005; Mungov et al. 2013). Their coverage is sparse, and they are difficult and expensive to maintain.

Seismic data may be used to constrain the strength of a potential tsunami. Inverting the available data and using existing fault models, one can obtain an approximate source moment tensor and in turn an approximate seafloor uplift, which can then be used as input for a shallow water model of the ocean. Aside from the uncertainties of the aforementioned steps, there is also in general no clear correlation between tsunamigenic potential and radiated seismic energy–especially for shallow megathrust events (Manta et al. 2020). For instance, the 2010 Mentawai earthquake had a rather small surface wave magnitude, but caused a large tsunami because of its shallow focal depth and slow fault rupture. This introduces uncertainties into tsunami warning systems that only use seismic data.

A number of international tsunami warning centers aim to provide early warning to coastal communities (Bernard and Meinig 2011; Falck et al. 2010; Whitmore et al. 2008). They usually combine a rapid inversion of the seismic data and/or local ground displacement measurements, typically through GNSS, and sometimes in real time (Kawamoto et al. 2016; Kawamoto et al. 2017). This approach is the fastest strategy possible for near-field warnings, but cannot accurately model the far-field propagation of a tsunami. The Science Monitoring And Reliable Telecommunications (SMART) cables (Howe et al. 2019) provide instruments on undersea fiber optic cables in order to increase the data coverage of the deep ocean. Such ocean bottom systems have already proved capable of recording seismic events and tsunamis (Shinohara et al. 2021). However, systematic oceanic coverage along these lines is not yet available and would require an unrealistic scale of deployment. Japan’s DO-NET (Kawaguchi et al. 2015) and SNET (see https://doi.org/10.17598/nied.0007 and (Nishikawa et al. 2019)) are examples of existing seafloor infrastructures; they are deployed near the land–sea boundaries where most earthquakes occur, but might lack the visibility necessary to study the far-field propagation of tsunamis.

GNSS-based ionospheric monitoring overcomes most of these limitations. First, extremely large data volumes are already available at no additional cost, thanks to (1) the multiple satellite constellations orbiting earth and (2) the plethora of ground-based GNSS receivers spread around the globe. Ionospheric measurements can typically be obtained up to 1200 km (assuming a 15º elevation cutoff and an ionosphere at 350 km altitude) from a given ground station, ensuring excellent spatial coverage. Waves traveling in the ionosphere are a direct proxy for the characteristics of the event that generated them, and the inversion of wave parameters is particularly straightforward for simple tsunami waves. Finally, near-real-time (NRT) TEC analyses can be performed within minutes of the atmospheric wave reaching the ionosphere. All these arguments make NRT GNSS-based monitoring of the ionosphere an attractive augmentation to natural hazards EWS.

A convenient method of obtaining TEC time series from GNSS data in post-processing (as opposed to real time) is to process Receiver Independent Exchange (RINEX) files. Typically, a file contains data for one station for a given day, and stations are managed by various agencies, universities, or research institutions. The data are made available through various organizations such as the International GNSS Service (IGS, http://www.igs.org) or the University Navstar Consortium (UNAVCO, http://www.unavco.org) or through country- or university-specific services.

There already exist a number of NRT ionospheric products. However, most of them are aimed at space weather applications and mitigation, and therefore only provide ionospheric maps at cadences that are too low for early warning systems. The IGS provides global ionospheric maps at various rates (Hernández-Pajares et al. 2009) (https://igs.org/products/#ionospheric_products), including in NRT (Liu et al. 2021) (for now available at http://chapman.upc.es/irtg/last_results/). SIMuRG (Yasyukevich et al. 2020) (https://simurg.iszf.irk.ru/) uses multiple international networks to provide daily and upon-request ionospheric products, such as an approximately bias-free absolute TEC, filtered differential TEC, and various indices. IONORING (Cesaroni et al. 2021) (http://ionos.ingv.it/ionoring/ionoring.htm) provides NRT ionospheric maps over Italy by utilizing data from the national Italian GPS network (RING, Rete Integrata Nazionale GPS). The European Space Agency’s (ESA’s) Space Weather Service Network (Kruglanski et al. 2015) provides NRT ionospheric maps over Europe and globally, among other products related to space weather. Finally, we note that Savastano et al. developed an early version of a NRT ionospheric monitoring system, which relied on the VARION algorithm (Savastano 2018; Savastano et al. 2017) from the VADASE software (Benedetti et al. 2015).

In this work, we focus on JPL’s Global Differential GPS (GDGPS) network, which collects and processes multi-GNSS data from hundreds of globally distributed stations, including a significant selection of locations along the Pacific Ring of Fire. The data are transferred in BINEX format at a 1 Hz cadence in NRT. The objective of the GUARDIAN system is to use JPL’s real-time GDGPS capabilities to develop an NRT ionospheric monitoring system for natural hazards.

We describe the GNSS Upper Atmospheric Real-time Disaster Information and Alert Network (GUARDIAN). We first describe the GUARDIAN architecture. The following sections present the elements of that architecture: the delivery of data to the GDGPS server, the general buffering scheme allowing for near-real-time operations, the mitigation of data issues and the computation of the TEC, and the publicly available web page. We then compare sample data obtained through our real-time system and computed offline. Finally, we describe future steps in improving the GUARDIAN, and conclude with overview on our system.

## System architecture

Figure 1 presents the general architecture of GUARDIAN. Real-time multi-GNSS carrier phase data and satellite orbits are collected in the first module and buffered. These data are then used to compute TEC and Ionospheric Pierce Points (IPPs); see (1.1) in Fig. 1, and detailed description in the “Total Electron Content and Ionospheric Pierce Points Computation” section. The resulting data are regularly output to files for further analysis; this is presented in sections “Real-Time Buffer, Output, and Filter,” “Data Conditioning,” and “Total Electron Content and Ionospheric Pierce Points Computation.” The computed data are first and foremost displayed on a publicly accessible web page, https://guardian.jpl.nasa.gov. See the “Public Web Interface” section and the “Public Web Interface” block in Fig. 1.

The planned architecture will also include detection and inversion systems (see (2) and (3), respectively, in Fig. 1). The goal of those two blocks is to provide an automated warning based on the TEC data stream. The first-level warnings would only rely on automated detections. A confirmation would be derived from the inversion, as well as more detailed information on the potential event if applicable. Both warnings would be displayed on the web page and could be interfaced with existing EWS through an Application Programming Interface (API).

### GNSS networks and GDGPS data delivery

The current version of GUARDIAN uses data from the GPS, Galileo, GLONASS, and BDS constellations. In RINEX-3 terminology, the observation codes used are (L1C, L2W) for GPS, (L1C, L2C) or (L1P, L2P) for GLONASS, (L1C, L5Q) or (L1X, L5X) for Galileo, and (L2I, L6I) or (L2I, L7I) for BDS. Currently, more than 80 stations along the coast of the Pacific Ocean are included in the network. However, this list can readily be extended up to a total of more than 200 stations processed by JPL’s GDGPS, at the only cost of more computational power.

JPL’s GDGPS network collects raw carrier phase and pseudorange 1 Hz tracking data from a set of stations in real-time. Depending on the remote station, the data are transmitted over Internet Protocol (IP) with either a BINEX (Binary RINEX) or RTCM-3 format to GDGPS servers.

Next, at a GDGPS server dedicated to GUARDIAN processing, data are eventually selected according to data type and a subset list of stations. A GNSS Data Editor (GDE) recently developed at JPL, ABD (Advanced Break Detector, developed mainly by B. Szilágyi), then separates arcs in the data, flags disjointed arcs, and attempts to correct cycle slips; see (Blewitt 1990) for more details on the typical process used in GDEs. Finally, these data samples are pushed to a shared memory slot in the memory of the dedicated GUARDIAN GDGPS’ server, allowing other processes to access it.

Following the same rationale, multi-GNSS orbits are also collected in real time and accessible directly on the dedicated server, allowing us to compute IPPs in real time as well (see the “TEC and IPP Computation” section).

Note that this base architecture is also used by other NASA/JPL applications. The GPS Real Time Earthquake and Tsunami (GREAT) alert system utilizes precise point positioning (PPP) to retrieve co-seismic site motions and therefore assess the parameters of large earthquakes and the eventual resulting tsunamis (Bar-Sever et al. 2010). The GPS-Aided Tsunami Early Detection (GATED) utilizes near-field (epicentral distance < 1000 km) GNSS data and mid-range (30º < epicentral distance < 45º) teleseismic P-waves to invert earthquake mechanisms in real time, with the goal of augmenting tsunami early warning systems (Chen et al. 2020). Another application is the real-time integrity monitoring of GNSS constellations.

This data delivery scheme is also easily reproducible, both for phase data and orbits. For instance, Maletckii and Astafyeva (2021) also propose to use the Networked Transport of RTCM (Radio Technical Commission for Maritime Services 2020) via Internet Protocol (NTRIP) (ESA GNSS Science Support Centre 2018).

### Real-time buffer, output, and filter

We use buffering for two main reasons. First and foremost, buffering allows us to use advanced tools to correct and compute the quantities of interest in an NRT time frame, consistent with our overarching NRT early warning goal. Second, hardware considerations limit the rate at which data points can be output to files. Consequently, since we need to keep up with the NRT data rate, we need to limit the frequency of the computationally heavy file operations (open, write, and close).

For each new satellite–station link, a buffer is initialized. Its length is parametrizable and is set to 10-min in the current implementation (based on empirical stability testing). The buffer keeps track of six fields: time, both carrier phases, and satellite positions in ECEF (Earth Centered Earth Fixed) convention. We progressively fill the buffer by continuously fetching data from the real-time GDGPS memory (carrier phase and orbits). When a link’s buffer has been filled, we proceed with corrections, TEC computation, IPP computation, and writing to disk.

First, cycle slips and data gaps are corrected; this is detailed in the “Data Conditioning” section. Next, we compute the TEC using the carrier phase time series, and the IPPs using the ECEF satellite and ground station coordinates; this is detailed in the “TEC and IPP Computation” section. Finally, the TEC product is output to the relevant file. The file structure consists of one file per station, containing observations for all available satellites.

The resulting time series need to be filtered before being displayed on the website. The current system implements a fourth-order Butterworth high-pass filter with cutoff period 15 min (about 1.1 mHz), allowing the observation of gravity waves while filtering out planetary waves, the diurnal TEC variation, and other large-scale phenomena.

Note that the data-collecting backend outputs absolute (unfiltered), uncalibrated TEC time series to files. The filtering occurs through a separate process, which is scheduled to happen closely after each buffer is output. Consequently, the filtering is able to account for whole arcs, while still taking into account the most recent data possible. This allows us to use the necessarily low cutoff frequency without encountering any data analysis issues. For instance, filtering a 5-min-long time series with a high-pass filter with a 10-min cutoff period is intrinsically unstable; the method we employ circumvents this issue.

It is useful to note that some particular ionospheric disturbances may still remain in the data after filtering (sudden increases in TEC following coronal mass ejections, sporadic E irregularities, etc.)—it would, however, not be difficult to discriminate them from disturbances originating from the hazards-induced neutral atmosphere forcings of interest here.

### Data conditioning

Cycle slips (CSs) are usually caused by a temporary loss of lock in carrier tracking; as a result, the observed phase skips one whole wavelength (or more), manifesting as a nonphysical jump in the phase measurement. JPL’s GDE flags disjointed arcs and associated CSs, and attempts to correct as many of them as possible.

Eventual in-arc CSs are detected using a modified Z-score-based outlier criteria (described in Appendix) on the first-order differential of each of the carrier phase measurements. The first-order differentiation allows a better localization in time of the eventual CSs. The detected CSs are then fitted using a Heaviside-augmented polynomial and corrected if the jump (the Heaviside coefficient in the fit) is an integer multiple of the wavelength at play.

We note that more advanced CS fixing methods exist (Cai et al. 2013; Hofmann-Wellenhof et al. 2008; Wu et al. 2010; Zangeneh-Nejad et al. 2017); their implementation is, however, deferred to future work.

Because real-time data collection methods are subject to transmission errors and packet losses, our data stream is subject to potential data gaps. It is impractical to slow down the system to wait for those missing samples to eventually be received. Rather, we implement a mitigation strategy described below.

Since samples are time-stamped, missing samples can be spotted at each shared memory fetch step during the buffering process, allowing us to flag data gaps. At the output step, the points flagged as missing are interpolated using the two neighboring data segments. We keep these interpolated points flagged for subsequent steps in our system.

When the elevation is too low (lower than approximately 7º), when the link is losing lock, or if there is a hardware or network issue, the number of gaps might surge. The currently buffered data is hence considered unreliable and needs to be discarded; we empty it and restart a fresh buffer with the latest sample. We implement two empirical discard thresholds: (1) if a single gap is longer than 15% of the total number of points in the buffer, or (2) if the total number of missing points in the buffer is greater than 50% of the total number of points in the buffer.

### Total electron content and ionospheric pierce points computation

Excluding nondispersive terms in the carrier phase GNSS observables, the carrier phase difference is driven by the integral of the radio signals’ refractive index along the signal path (Teunissen and Montenbruck 2017, Eq. (6.83)) and is derived using the Appleton–Hartree formula for this refractive index in the ionospheric plasma (Teunissen and Montenbruck 2017, Eq. (6.72)). As a result, the single difference of dual-frequency measurements is directly proportional to the TEC along the receiver–transmitter (RT) line of sight:

$$\phi_{{f_{1} }} - \phi_{{f_{2} }} = \frac{{K\left( {f_{1}^{2} - f_{2}^{2} } \right)}}{{f_{1}^{2} f_{2}^{2} }}{\mathbb{S}} + {\Delta }b + {\Delta }\varepsilon$$

where $f_{1,2}$ are the considered carrier frequencies, $\phi_{{f_{1} ,f_{2} }}$ are the measured phases along the respective frequencies, ${\mathbb{S}}$ is the slant TEC (STEC, the integral of the electron density along the slant ray path), ${\Delta }b$ contains instrumental or interfrequency delays (station and satellite), and ${\Delta }\varepsilon$ contains residual noise terms (e.g., local multipath and thermal noise). $K$ is a constant derived from the plasma frequency (Teunissen and Montenbruck 2017, Eq. (39.3)):

$$K = \frac{1}{2}\frac{{q_{{\text{e}}}^{2} }}{{4\pi^{2} \varepsilon_{0} m_{{\text{e}}} }} \approx 40.308193 {\text{m}}^{3} {\text{s}}^{ - 2}$$

where $q_{{\text{e}}}$ is the electron charge, $\varepsilon_{0}$ is the dielectric constant of vacuum, and $m_{{\text{e}}}$ is the electron mass. ${\Delta }b$ and ${\Delta }\varepsilon$ are considered higher-order terms. As a result, to first order, ${\mathbb{S}}$ can be estimated through:

$${\mathbb{S}} \approx \frac{{f_{1}^{2} f_{2}^{2} }}{{K\left( {f_{1}^{2} - f_{2}^{2} } \right)}}\left( {\phi_{{f_{1} }} - \phi_{{f_{2} }} } \right)$$

The retrieved TEC is absolute and uncalibrated, and, in practice, the instrumental delays ${\Delta }b$ induce STEC amplitude shifts, which can, however, be assumed constant for each RT couple over several days (Mannucci et al. 1998, and references therein). Algorithms exist to estimate and correct those biases (Bertiger et al. 2020; Bertiger et al. 2010; Blewitt 1989; Odijk and Teunissen 2013; Vierinen et al. 2016), but are impractical to apply in real time. However, this does not introduce additional complexities on GUARDIAN’s detection scheme because the filtering process removes any unestimated biases from the TEC time series.

For a given satellite–station link, it is customary to define the point in space where the TEC estimate is valid as the IPP for this measurement. Here, we define the IPPs as the intersection between the satellite–station line of sight and a single-shell ionospheric model fixed at 350 km altitude. We compute the IPPs through an iterative fixed-point algorithm.

We note that a fixed single-shell model might not be exact in all possible conditions due to the fact that the ionosphere is a highly dynamic environment. A different shell height would not significantly displace the IPPs, but may have a slight impact on the determination of the celerity and direction of propagation for TIDs (Komjáthy 1997). However, introducing shell models varying in time and/or space would adversely impact the consistency of our products in time and space. In short, we deem that the potential errors introduced by our choice of shell model are negligible for this current work.

### Public web interface

Data processed by the python-based GUARDIAN backend system are displayed in near-real time on an interactive web interface at https://guardian.jpl.nasa.gov. Data generated in CSV (Comma-Separated Values) format by the backend system are converted to JSON (JavaScript Object Notation) files for web plotting and manipulation by a converter script, which is run automatically every 6 min. The user arrives at the GUARDIAN website, where a map with GDGPS stations (represented by black squares), earthquakes (represented by yellow circles), and IPPs (represented by colored streaks) is displayed. Only the last 60 min of IPP data are shown on the map. Color-coded circles indicate earthquakes flagged as potentially tsunamigenic by the United States Geological Survey (USGS). A selection rectangle may be dragged on the map, which populates a sidebar with earthquakes and stations within the square. Additional rectangles may be drawn to append to the sidebar. Hovering the mouse over a station or earthquake in the sidebar enlarges the feature on the map to highlight its location. Upon clicking an earthquake in the sidebar, additional details such as the location and time of the earthquake, are displayed below the map. Upon clicking any station, a window containing check boxes with satellite names is revealed. Each check box, when checked, plots slant TEC data from the GUARDIAN backend associated with that satellite and the clicked station over the last 24 h. The plot can be zoomed and dragged to study specific features in the TEC profile. Also plotted are locations of corrected cycle slips, which may inform the user of possible filtering artifacts that can be mistaken for geophysical signals.

### Real-time TEC validation

In this section, we verify that our real-time processing yields TEC time series comparable to the time series one could obtain by post-processing daily RINEX files. On the one hand, we record TEC time series in real-time using the GUARDIAN system. On the other hand, once the day of interest has passed, we obtain “ground truth” TEC time series by post-processing the corresponding GDGPS archive files with JPL’s proprietary Python software GNSSTEC.

**Fig. 2:**
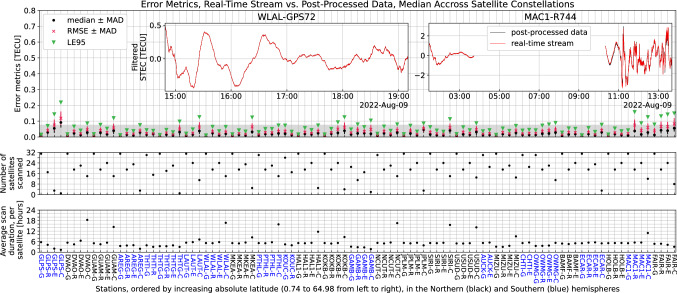
Validation of the real-time stream. See main text for a description of the dataset analyzed here. Top panel: various error metrics for each station–constellation couple. See main text for a description of each metric. The gray bar corresponds to the range of observed noise levels in this dataset: typically, below 0.077 TECU. Top panel, insets: The left inset represents the best link in terms of error metrics (in TECU, LE95 = 0.005, median error = 0.001, RMSE = 0.002, MAD = 0.001). The right inset represents a relatively bad link (in TECU, LE95 = 0.515, median error = 0.049, RMSE = 0.202, MAD = 0.098); 99% of all links summarized here have a better agreement than this link. Middle panel: number of satellites scanned, for each station–constellation couple. Bottom panel: average duration of the time series used for computing the error metrics, per satellite, for each station–constellation couple. For instance, for the station–constellation couple GLPS-G, 31 satellites were scanned, with an average scan duration of 5.7 h per satellite; thus, for this couple, the error metrics in the top panel are computed with data spanning 177.75 h

Figure 2 summarizes the validation for 24 h of data acquired on August 9, 2022. We make sure to analyze a significant sample of stations around the Pacific Ring of Fire, both in the northern and southern hemispheres and at the widest range of magnetic latitudes. This plot summarizes 1987 links recorded over 27 stations in the Pacific (13 in the northern hemisphere and 14 in the southern hemisphere), covering absolute latitudes from 0.74 to 64.98. The cumulative duration of all time series summarized here amounts to 10,876 h.

The data for computing the error metrics (see below) and for displaying the insets has been filtered with a zero-phase fourth-order Butterworth high-pass filter; for computing the metrics, the cutoff was chosen at 0.28 mHz (periods < 60 min); for displaying the insets, the cutoff was chosen at 3.33 mHz (periods < 5 min).

The error was computed as the absolute error between the post-processed data and the real-time stream. Based on these error time series, we computed four metrics to evaluate the agreement between the real-time stream and the post-processed data:

- LE95, the 95-%-quantile of absolute errors.
- median, the median of absolute errors along the time series.
- RMSE, the root-mean-squared of the absolute error.
- MAD, the median absolute deviation of the absolute errors, computed link-wise; MAD = median(|absolute error—mean absolute error|); we use it as a metric for the dispersion of the absolute error and add it as a bar around both the mean and RMSE metrics.

It is to be noted that, in order to reduce the metrics to a single value per station–constellation couple in Fig. 2, we computed the median of each metric across each constellation. Notice also that GLPS-E and GLPS-C only display 3 and 1 satellite respectively; these errors are slightly worse than the rest of the links due to issues with underlying data gaps on those 4 links.

In summary, we note that 97% of the links checked showcase an RMS error below 0.1 TECU, and 86% of them verify RMSE < 0.05 TECU (the typical noise level for carrier phase measurements). The main source of errors stems from data quality issues shortening the effective length of the arcs to be filtered: data gaps, or discontinuous arcs due to uncorrected cycle slips. These points are subject to continuous work as part of our efforts to maintain and improve the GUARDIAN system. In short, we deem the GUARDIAN near-real-time stream validation sufficient for all intents and purposes at this stage.

### Future work: automated detection

At present, the GUARDIAN system displays data that can be explored manually by a subject matter expert, who can identify tsunamigenic signatures in TEC time series in the vicinity of a potentially tsunamigenic earthquake. The next step in our system is automatically detecting perturbations in the TEC time series. Ideally, we aim to capture and flag every single anomalous perturbation. Based on previous studies (Afraimovich et al. 2001; Artru et al. 2005; Astafyeva 2019; Astafyeva et al. 2014; Lay et al. 2015; Liu et al. 2019; Maletckii and Astafyeva 2021; Manta et al. 2020, 2021; Occhipinti 2015; Occhipinti et al. 2013; Sanchez et al. 2022), we expect to be able to detect ionospheric disturbances due to earthquakes, tsunamis, volcanic eruptions, and storms, as well as space weather effects.

Several techniques for automated detections already exist in the literature, for instance: ionospheric power indices (Manta et al. 2020, 2021), wavelet analysis threshold-based contouring (Torrence and Compo 1998), 2D principal component analysis (Lin 2021), random forests (Brissaud and Astafyeva 2022), or Gramian angular fields (Constantinou et al. 2021). Those will be explored in future work based on the real-time TEC streams presented in this work.

### Future work: Tsunami parameter inversion

A key component of tsunami EWS (TEWS) is the capability to infer the properties of a detected tsunami as it travels toward coastal regions. By inverting the tsunami-induced TEC perturbations using a normal-modes summation model, Rakoto et al. (2018) were the first to infer tsunami wave heights, achieving a 20% accuracy for three events (2006 Kuril Islands, 2011 Tōhoku-Oki, and 2012 Haida Gwaii).

**Fig. 3:**
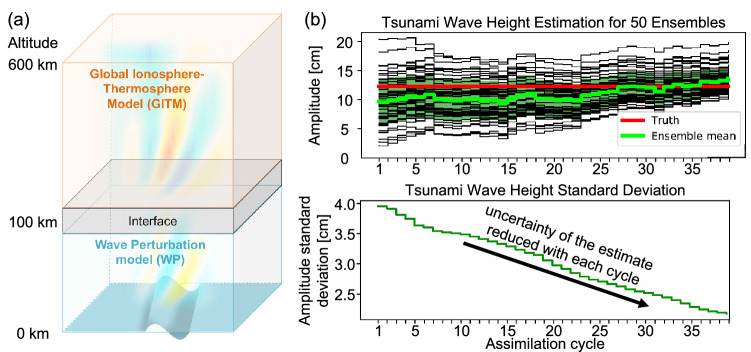
Proof of concept of the tsunami parameter inversion using synthetic TEC data. **a** Schematic of the physical space modeled by WP-GITM (reproduced with permission after (Komjáthy et al. 2016)). WP models the propagation of waves in the neutral atmosphere, while GITM simulates the thermospheric and ionospheric effects. **b** Tsunami wave height inversion from modeled ionospheric disturbances, for the case of the 2011 Tōhoku-Oki tsunami. (**b**), top: Tsunami wave height inversion as a function of assimilation cycle using DART/WP-GITM. In this case, each assimilation cycle is 5 min. The solid black lines show the ensemble members of the data assimilation, the solid green line shows the ensemble mean state, and the solid red line shows the “true” tsunami wave height from the MOST model. (**b**), bottom: Time evolution of the standard deviation of the ensemble means, as a function of the assimilation cycle

We have developed a prototype software that infers tsunami wave heights with 10% accuracy by inverting simulated electron density perturbations. This new software, named DART/WP-GITM, although at an experimental stage, is a joint JPL/NCAR effort based on ensemble data assimilation driven by 3D physics-based tsunami–ionosphere coupling model simulations. DART is publicly available at https://dart.ucar.edu and is described in detail by Anderson et al. (2009). GITM is publicly available at https://github.com/aaronjridley/GITM (Ridley et al. 2006), and Meng et al. (2015) describe its application for simulating the Tōhoku-Oki event. The novelty of DART/WP-GITM is the development of an interface between these two existing pieces of software, where DART assimilates the WP-GITM simulations. The general working principle is illustrated in block (3) of Fig. 1, an illustration of the modeled domain is given in Fig. 3a, and a detailed description is given in Supplementary Text T1. We applied this prototype software to the 2011 Tōhoku-Oki tsunami (https://earthquake.usgs.gov/earthquakes/eventpage/official20110311054624120_30). Details of the assimilation procedure are given in Supplementary Text T2. Each assimilation time step lasted about 5 min, for a total run-time of 3 h. Figure 3b (top) shows the convergence of the initial ensemble tsunami states (solid black lines) and its mean (solid green line) to the “true” MOST-estimated tsunami wave height (solid red line) as a function of the assimilation cycle. The reduction in the ensemble spread together with the reduction of the standard deviation of the inverted tsunami wave height by 50% at the end of the simulation time to 2.0 cm (see Fig. 3b, bottom) is consistent with a successful tsunami wave height inversion. Although our inversion does not converge to ± 2.0 cm to the “truth” until after 3 h of simulations, the results are encouraging in terms of detecting open ocean tsunamis traveling toward the US west coast.

We envision augmenting the GUARDIAN system with this experimental prototype by initiating a DART/WP-GITM inversions on automatically-detected ionospheric perturbations in order to estimate tsunami wave properties. Significant software developments are, however, still required to test DART/WP-GITM with real GNSS satellite observations.

We note that the inversion technique presented in this section relies on the TIDs induced by the internal atmospheric gravity waves (IAGWs), themselves induced by the propagating tsunami. Due to slower phase velocities, IAGWs take longer to propagate from the ocean surface to the ionosphere, typically on the order of 30–40 min (Astafyeva 2019). As a result, IAWG-induced TID-based inversions are only practical for far-field applications for which the tsunami travel time exceeds the atmospheric propagation time.

Because of much shorter tsunami travel times in the near-field, risk assessments must rely on the acoustic waves (AWs) launched from the initial sea surface uplift. Such AWs reach the ionosphere typically within 8–10 min (Astafyeva 2019), and the TEC perturbations they induce are directly proportional to the magnitude of the event (see (Heki 2021; Manta et al. 2020; and references therein)).

## Conclusion

In this work, we have introduced the first near-real-time ionospheric monitoring network, GUARDIAN. Leveraging JPL’s GDGPS network, we focus on the Pacific Ring of Fire, with the goal of monitoring natural hazards in this seismic/volcanic highly active region. At the time of publication, 53% of the total area of the region of interest in the Pacific is monitored by the GUARDIAN system (see Supplementary Figure F1). This corresponds to almost two-thirds of the maximum possible coverage (82%). Implementing additional stations in strategic locations will allow to increase this coverage further.

The data collection system is now fully functioning and validated (see the “Real-Time TEC Validation” section), as well as publicly accessible to the general online community (Sect. 6, https://guardian.jpl.nasa.gov).

The DART/WP-GITM inversion scheme for tsunami waves is currently working as standalone (see the “Future Work: Tsunami Parameter Inversion” section). In future work, we seek to link this method within the GUARDIAN architecture (Fig. 1). Furthermore, the overarching goal would be to develop additional inversion tools geared for other natural hazards (volcanic eruptions, storms, etc.).

Our next step will be implementing automatic detection algorithms to pinpoint TEC perturbations of interest for either tentative automatic inversions or manual investigation by analysts. Furthermore, by optimizing our near-real-time run-time and leveraging parallelization, we will extend our coverage to the whole set of stations available to GDGPS over the entire globe.

When investigating ionospheric perturbations caused by natural hazards, one has to distinguish between near-field and far-field applications. These two types of applications have different limitations, but the NRT monitoring enabled by GUARDIAN may be useful for both.

In the near-field, the natural hazards’ atmospheric perturbations will reach the ionosphere within approximately 10 min, making the near-real-time TEC analysis particularly valuable to assess the magnitude of the event. GUARDIAN is able to produce TEC time series within 10 min of the wave reaching the ionosphere, making our product the fastest TEC product available. We, however, note that atmospheric perturbations may reach the ionosphere as early as 8 min after an event (Astafyeva 2019; Astafyeva et al. 2011; Thomas et al. 2018; Yang et al. 2014), which would make GUARDIAN slightly late. Optimization of the buffer length and of our procedures will help reduce this lag, but we defer this to future work.

The GUARDIAN system is also valuable for far-field applications. For instance, we have shown it is possible to estimate wave parameters by inverting the tsunami-induced TEC perturbations (Sect. 9). This will, in the future, make our NRT product the earliest direct characterization of the sea surface height, which will in turn be used as initial conditions for tsunami propagation models.

## Appendix

### Acronyms

The following acronyms are used throughout this paper: API (Application Programming Interface), CSV (Comma-Separated Values), DART (Deep-ocean Assessment and Reporting of Tsunamis (Bernard and Meinig 2011; Meinig et al. 2005; Mungov et al. 2013), or Data Assimilation Research Testbed (Anderson et al. 2009)), ECEF (Earth Centered Earth Fixed, a reference frame), EWS (Early Warning System), GDE (GNSS Data Editor), GDGPS (JPL's Global Differential GPS network), GITM (Global Ionosphere Thermosphere Model), GNSS (Global Navigation Satellite System), GGOS (Global Geodetic Observing System), GREAT (JPL's GPS Real Time Earthquake and Tsunami), GTEWS (GNSS-based Tsunami Early Warning System), GUARDIAN (GNSS Upper Atmospheric Real-time Disaster Information and Alert Network), IAG (International Association of Geodesy), IGS (International GNSS Service), IPP (Ionospheric Pierce Point), IUGG (International Union of Geodesy and Geophysics), JPL (NASA’s Jet Propulsion Laboratory), NASA (National Aeronautics and Space Administration), NRT (Near-Real-Time), NTRIP (Networked Transport of RTCM via Internet Protocol), PPP (Precise Point Positioning), RINEX (Receiver Independent Exchange, a format), RTCM (Radio Technical Commission for Maritime Services), STEC (Slant TEC), TEC (Total Electron Content), TEWS (Tsunami Early Warning System), UNAVCO (University Navstar Consortium), WP-GITM (Wave Perturbation Global Ionosphere Thermosphere Model (Meng et al. 2015)).

## Appendix: Modified Z-score outlier detector

The modified Z-score is a type of outlier detector (Iglewicz and Hoaglin 1993). It is defined for a series $\left( {y_{i} } \right)_{i \in 1,N}$ as:

$$\forall i \in 1,N, Z_{i} = \left\{ {\begin{array}{*{20}l} {\frac{{y_{i} - \overline{y}}}{{\sigma_{{\overline{y}}} }}} \hfill & {{\text{if}}\;\sigma_{{\overline{y}}} \ne 0} \hfill \\ 1 \hfill & {{\text{if}}\;\sigma_{{\overline{y}}} = 0} \hfill \\ \end{array} } \right.$$

where the median $\overline{y}$ and the median absolute deviation $\sigma_{{\overline{y}}}$ are defined as:

$$\left\{ {\begin{array}{*{20}l} {\overline{y} = {\text{median}}\left( {\left( {y_{i} } \right)_{i \in 1,N} } \right)} \hfill \\ {\sigma_{{\overline{y}}} = {\text{median}}\left( {\left( {\left| {y_{i} - \overline{y}} \right|} \right)_{i \in 1,N} } \right)} \hfill \\ \end{array} } \right.$$

For more flexibility, we replace the median over the whole series $\overline{y}$ with a sliding median over $2k + 1$ elements around $i$, $\overline{y}_{i}^{k}$. We adapt the median absolute deviation accordingly. In fine, we define the sliding modified Z-score $Z_{i}^{k}$ as:

$$\forall i \in 2,N,\left\{ {\begin{array}{*{20}l} {Z_{i}^{k} = \left\{ {\begin{array}{*{20}l} {\frac{{y_{i} - \overline{y}_{i}^{k} }}{{\sigma_{{\overline{y}_{i}^{k} }} }}} \hfill & {{\text{if}}\;\sigma_{{\overline{y}_{i}^{k} }} \ne 0} \hfill \\ 1 \hfill & {{\text{if}}\;\sigma_{{\overline{y}_{i}^{k} }} = 0} \hfill \\ \end{array} } \right.} \hfill \\ {\overline{y}_{i}^{k} = {\text{median}}\left( {\left( {y_{i} } \right)_{i \in i - k,i + k} } \right)} \hfill \\ {\sigma_{{\overline{y}_{i}^{k} }} = {\text{median}}\left( {\left( {\left| {y_{i} - \overline{y}_{i}^{k} } \right|} \right)_{i \in 1,N} } \right)} \hfill \\ \end{array} } \right.$$

Following Iglewicz and Hoaglin (1993), we deem elements $i$ having a $Z_{i}^{k}$ higher than a detection threshold of $Z_{{{\text{max}}}}$ = 3.5/0.6745 ≃ 5.19 to be outliers.

## Supplementary Information

Below is the link to the electronic supplementary material.

Supplementary file1 (PDF 1757 kb)
