# Supplementary material for: The GUARDIAN system-a GNSS upper atmospheric real-time disaster information and alert network
Source: GPS Solut. 2022 Dec 3;27(1):32. doi: 10.1007/s10291-022-01365-6 (PMC9719453; doi:10.1007/s10291-022-01365-6)
Supplement: Supplementary file 1 — Supplementary file1 (PDF 1757 kb) [file 10291_2022_1365_MOESM1_ESM.pdf]

Supplementary Information for "The GUARDIAN System - a GNSS Upper Atmospheric Real-time Disaster Information and Alert Network"

Léo Martire[1*], Siddharth Krishnamoorthy[1], Panagiotis Vergados[1], Larry J. Romans[1], Béla Szilágyi[1], Xing Meng[1], Jeffrey L. Anderson[2], Attila Komjáthy[1], Yoaz E. Bar-Sever[1]

[1] Jet Propulsion Laboratory, California Institute of Technology, 4800 Oak Grove Drive, Pasadena, 91109, CA, USA.

[2] National Center for Atmospheric Research, 1850 Table Mesa Drive, Boulder, 80305, CO, USA.

[*] Corresponding author: leo.martire@outlook.com.

Contents

- Supplementary Text T1.
- Supplementary Text T2.
- Supplementary Figure F1.

Supplementary Text T1

The Data Assimilation Research Testbed (DART) (https://github.com/NCAR/DART, (Anderson et al. 2009)) and Wave Perturbation Global Ionosphere Thermosphere Model (WP-GITM) model (https://github.com/aaronjridley/GITM, (Meng et al. 2015)) are open-source software and publicly available. The DART/GITM interface that we designed uses an Ensemble Adjustment Kalman Filter (EAKF) to estimate the posterior states of WP-GITM and its external drivers (in this case tsunami properties) given an ensemble of prior model forecasts and a set of noisy (in this case synthetic) observations and their error variances.

Supplementary Text T2

Focusing on the 2011 Tōhoku-Oki tsunami (https://earthquake.usgs.gov/earthquakes/eventpage/official20110311054624120_30), we simulated the 3D state of the ionosphere when the tsunami was approaching the West coast of the US from 14:00 UTC to 17:20 UTC, and defined synthetic observations for the DART/WP-GITM experiment. Next, we generated normal distributions for the tsunami wave height and period, using the Method Of Splitting Tsunami (MOST) model estimates as the "truth". We randomly selected a value for the tsunami wave height and period from each distribution to generate an ensemble of 50 tsunami states with a large enough spread around the "truth", excluding the "true" values. Initializing WP-GITM with each ensemble tsunami state, we produced an ensemble of 50 3D modeled states of the ionosphere containing different tsunami responses. We initialized DART with the 50 ensemble states to infer the posterior mean 3D state of the ionosphere and the tsunami wave height/period with estimated errors. Each assimilation time step (WP-GITM and data initialization for the DART assimilation) was about 5 min, over a total run-time of 3 hours. At the end of each time step, the 50 ensemble WP-GITM states and the tsunami parameters were the priors for the next time step assimilation cycle. A total of 2000 3D states of the ionosphere and tsunami parameters were estimated with WP-GITM/DART running on NASA-Ames' Pleiades supercomputer.

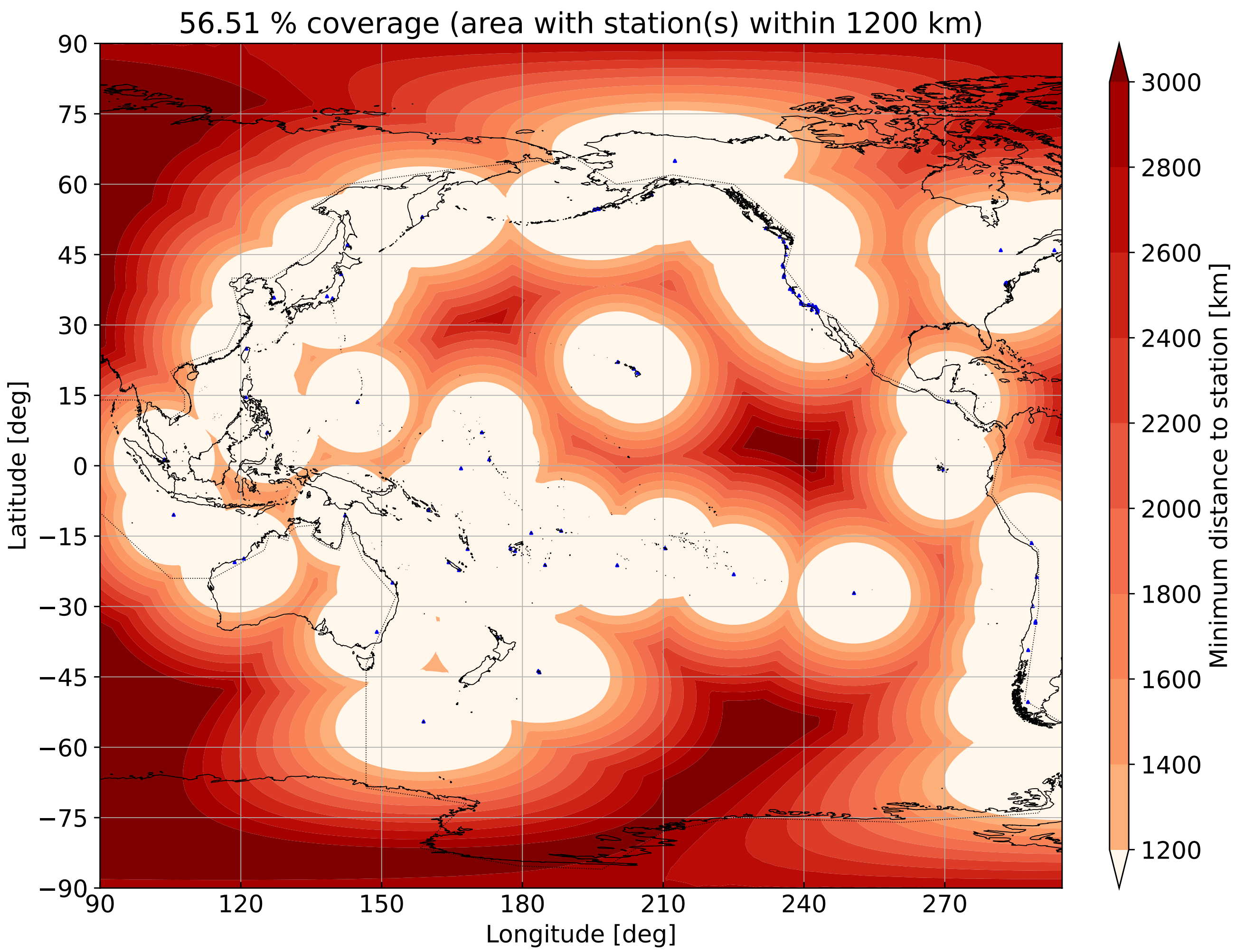

Fig. F1 Coverage of the ionosphere provided by the GDGPS stations currently monitored by the GUARDIAN system. The fine dotted line is a custom boundary the authors choose as being the geographic area of interest: the Pacific Ring of Fire, including the easternmost part of the Indian Ocean, to monitor risks linked to the Sumatran subduction zone. Note also that some stations in the GUARDIAN system monitor areas outside of the Pacific potentially subject to meteotsunamis (e.g., the U.S. East Coast). The 1200 km range limit is approximately equivalent to a 15° elevation cutoff if one assumes a single shell ionosphere at 350 km altitude; data may be acquired further if the noise levels remain low and if the GNSS signal remains locked. The colored ranges above 1200 km highlight how far from any given station some areas are, and thus, how valuable an additional station would be to the GUARDIAN system. However, some of those isolated areas are devoid of any kind of land; other types of satellite-based probing methods could, however, be used to fill those voids.

References

Anderson, J., Hoar, T., Raeder, K., Liu, H., Collins, N., Torn, R., Avellano, A. (2009). The Data Assimilation Research Testbed: A Community Facility. Bulletin of the American Meteorological Society, 90(9):1283–1296. https://doi.org/10.1175/2009BAMS2618.1.

Meng, X., Komjáthy, A., Verkhoglyadova, O.P., Yang, Y.-M., Deng, Y., Mannucci, A.J. (2015). A new physics-based modeling approach for tsunami-ionosphere coupling. Geophysical Research Letters, 42(12):4736–4744. https://doi.org/10.1002/2015GL064610.
